# Eating behavior dimensions and 9-year weight loss maintenance: a sub-study of the Finnish Diabetes prevention study

**DOI:** 10.1038/s41366-023-01300-w

**Published:** 2023-05-06

**Authors:** Jutta Salmela, Hanna Konttinen, Raimo Lappalainen, Joona Muotka, Anne Antikainen, Jaana Lindström, Jaakko Tuomilehto, Matti Uusitupa, Leila Karhunen

**Affiliations:** 1 Department of Psychology, University of Jyväskylä, Jyväskylä, Finland; 2 Social Psychology, Faculty of Social Sciences, University of Helsinki, Helsinki, Finland; 3 Endocrinology and Clinical Nutrition, Kuopio University Hospital, Wellbeing Services County of North Savo, Kuopio, Finland; 4 Department of Public Health and Welfare, Finnish Institute for Health and Welfare, Helsinki, Finland; 5 Department of Public Health, University of Helsinki, Helsinki, Finland; 6 Saudi Diabetes Research Group, King Abdulaziz University, Jeddah, Saudi Arabia; 7 Institute of Public Health and Clinical Nutrition, University of Eastern Finland, Kuopio, Finland

**Keywords:** Weight management, Pre-diabetes, Obesity, Randomized controlled trials, Nutrition

## Abstract

**Background:**

Behavioral processes through which lifestyle interventions influence risk factors for type 2 diabetes (T2DM), e.g., body weight, are not well-understood. We examined whether changes in psychological dimensions of eating behavior during the first year of lifestyle intervention would mediate the effects of intervention on body weight during a 9-year period.

**Methods:**

Middle-aged participants (38 men, 60 women) with overweight and impaired glucose tolerance (IGT) were randomized to an intensive, individualized lifestyle intervention group (*n* = 51) or a control group (*n* = 47). At baseline and annually thereafter until nine years body weight was measured and the Three Factor Eating Questionnaire assessing cognitive restraint of eating with flexible and rigid components, disinhibition and susceptibility to hunger was completed. This was a sub-study of the Finnish Diabetes Prevention Study, conducted in Kuopio research center.

**Results:**

During the first year of the intervention total cognitive (4.6 vs. 1.7 scores; *p* < 0.001), flexible (1.7 vs. 0.9; *p* = 0.018) and rigid (1.6 vs. 0.5; *p* = 0.001) restraint of eating increased, and body weight decreased (−5.2 vs. −1.2 kg; *p* < 0.001) more in the intervention group compared with the control group. The difference between the groups remained significant up to nine years regarding total (2.6 vs. 0.1 scores; *p* = 0.002) and rigid restraint (1.0 vs. 0.4; *p* = 0.004), and weight loss (−3.0 vs. 0.1 kg; *p* = 0.046). The first-year increases in total, flexible and rigid restraint statistically mediated the impact of intervention on weight loss during the 9-year study period.

**Conclusions:**

Lifestyle intervention with intensive and individually tailored, professional counselling had long-lasting effects on cognitive restraint of eating and body weight in middle-aged participants with overweight and IGT. The mediation analyses suggest that early phase increase in cognitive restraint could have a role in long-term weight loss maintenance. This is important because long-term weight loss maintenance has various health benefits, including reduced risk of T2DM.

## Introduction

Type 2 diabetes mellitus (T2DM) is preventable by lifestyle interventions targeting weight reduction by changes in diet and physical activity [1, 2]. Meta-analyses of randomized controlled trials (RCTs) have, however, shown that a large proportion of people are unable to maintain achieved weight loss [3, 4]. Body weight is a result of a variety of factors, some of which are more and some less modifiable [5]. Therefore, to improve long-term weight loss maintenance, it is important to understand especially the modifiable factors and their role as potential mediators of successful weight loss maintenance. Such potential factors include psychological dimensions of eating behavior [6, 7].

As a distinction of eating habits describing actual food intake, we refer here by the term eating behavior to its psychological dimensions related to motivation to eat [7]. These eating behavior dimensions could influence food intake through choices about what, when and where to eat [7–9]. Different dimensions of eating behavior have been identified, such as cognitive restraint of eating, disinhibition and susceptibility to hunger [10]. Cognitive restraint of eating refers to tendency to restrict food intake to lose weight or prevent weight gain. It can be further divided to flexible restraint (a more graduated approach to eating, dieting, and weight) and rigid restraint (a more dichotomous, “all-or-nothing” approach to eating, dieting, and weight) [11]. Disinhibition refers to tendency to eat opportunistically in an obesogenic environment in response to a variety of food and eating stimuli (e.g. emotions, food cues), and susceptibility to hunger to extent of experiencing feelings of hunger and food cravings in different situations.

Longitudinal weight management intervention studies have shown that an increase in cognitive restraint of eating is associated with successful weight loss in participants with overweight or obesity [12–14] and with long-term (≥1.5 year) weight maintenance after weight loss [15–17]. Increases in both rigid and flexible restraint have been associated with weight loss [18]. Components of restraint of eating may also have divergent associations with health-related outcomes [19] and especially an increase in flexible restraint has been associated with larger weight loss and/or better weight loss maintenance [20, 21]. A reduction in disinhibition [12, 21] and susceptibility to hunger [22] have also predicted weight loss, at least short-term.

Studies that have investigated eating behavior dimensions among people with impaired glucose tolerance (IGT) are still few. The non-controlled Delay of Impaired Glucose Tolerance by a Healthy Lifestyle Trial (DELIGHT) showed that enhancing flexible control and decreasing disinhibition seemed beneficial to control central adiposity and blood glucose in a 1-year follow-up [23]. Increased dietary restraint measured by DEBQ (Dutch Eating Behavior Questionnaire) [24] predicted better long-term weight loss among people with IGT and elevated fasting plasma glucose after a mean follow-up of 2.8 years according to a non-controlled sub-study of the US Diabetes Prevention Program (DPP) [25, 26]. Only one previous intervention study has investigated eating behavior dimensions as mediators of weight loss and weight loss maintenance, although not specifically in those with IGT [27]. It used mediation models to identify mediators of 12-month weight loss and 24-month weight loss maintenance in women with overweight/obesity who underwent a 1-year behavioral treatment program. An increase in flexible, but not rigid, restraint mediated a greater 24-month weight loss.

Thus, more evidence is needed about the role of eating behavior dimensions in long-term weight loss maintenance among people at risk of T2DM. The aim of the present study was to investigate how lifestyle intervention to prevent T2DM affects eating behavior dimensions (cognitive restraint of eating with flexible and rigid components, disinhibition, and susceptibility to hunger) among people with IGT as well as whether changes in eating behavior dimensions during the early phase (i.e. first year) of the lifestyle intervention mediate the long-term effects of the intervention on body weight.

## Methods

### Participants and procedure

This is a sub-study to the Finnish Diabetes Prevention Study (DPS)—a multicenter lifestyle intervention RCT with a parallel design aiming at assessing the efficacy of lifestyle management to prevent or delay the onset T2DM in people with IGT [28–30]. The DPS study was registered with ClinicalTrials.gov (NCT00518167). In the present study we used the eating behavior data collected only in the Kuopio DPS research center, with 38 men and 60 women comprising the study group. People were recruited by screening of high-risk individuals or were identified in earlier epidemiological surveys. The main inclusion criteria were age 40–64 years, body mass index (BMI) > 25 kg/m^2^ and IGT based on the mean of two 75 g oral glucose tolerance tests (OGTT) (WHO 1985 criteria). At baseline, the mean (SD) age was 53.6 (7.4) years and BMI was 31.3 (4.6) (kg/m^2^). The study protocol was approved by the ethics committees of the National Public Health Institute in Helsinki, Finland (intervention phase), and of the North Ostrobothnia Hospital District (post-intervention follow-up period). All participants gave written informed consent at baseline and again at the beginning of the post-intervention follow-up.

The RCT started in November 1993 and the recruitment period lasted until June 1997. Randomization to the intervention (*n* = 51 in the present study) or the control group (*n* = 47 in the present study) was stratified by center, sex, and baseline 2-h plasma glucose to ensure a balanced study design [30]. All participants had a baseline measurement and annual examinations that included standardized questionnaires and clinical and laboratory measurements. The current study includes data from measurements until November 2006 (i.e. 9 years from the initiation of the study). Participants diagnosed with T2DM during the intervention phase were excluded from the rest of the intervention phase examinations. The intervention phase of the study was discontinued prematurely as recommended by the independent end point committee based on interim endpoint analyses showing that the original research question of the DPS had been reached earlier than had been anticipated [30]. Thus, the median duration of the intervention varied among study participants (median 5 and range 3–6 years in Kuopio) because of the lengthy recruitment period. At the last intervention period visit all participants were given a summary of their laboratory test results during the intervention period, and they were told about the findings of the trial [31]. A post-intervention follow-up was offered to all participants, both those who had not developed T2DM as well as those who had developed T2DM. This article presents the results from the intervention and post-intervention follow-up phases corresponding altogether nine years from baseline. A total of 24 participants did not complete the 9-year study period. Based on Mann–Whitney *U* test or *χ*^2^ test, they did not differ in any of the baseline characteristics from those (*n* = 74) who completed the entire period (Supplementary Table 1). The fifth-year annual study visit was missing from one participant and the seventh-year study visit from 13 participants.

#### Lifestyle intervention group

The specific intervention goals were weight reduction (5% or more from baseline weight), dietary modification (energy proportion of total fat <30% and saturated fat <10% of total energy and dietary fiber intake at least 3.6 g/MJ (15 g/1000 kcal), and moderate intensity physical activity (30 min/day or more) [29]. Lifestyle intervention included seven face-to-face consultation sessions (from 30 min to 1 h) with the study nutritionist during the first year and every 3 months thereafter for 3–6 years depending on intervention duration. The sessions in the first year had preplanned topics (e.g., diabetes risk factors, saturated fat, fiber, physical activity, and problem solving), but the discussions were individualized, focusing on specific individual problems. Printed material was used to serve as a reminder at home.

The goal of the counselling was to equip the participants with necessary skills and knowledge and to achieve gradual, permanent behavioral changes to prevent the progression of IGT to T2DM [29]. The dietary advice was tailored to each participant based on 3-day food records, which were filled out four times yearly. Calculated nutrient intakes and a summary of the results were given to the participants with further explanations. The spouse was invited to join some of the sessions, especially if he or she was the one responsible for cooking or shopping in the family.

Goal-setting was an integral part of the intervention. Participants were encouraged to establish personal intermediate lifestyle goals for themselves by proposing practical issues that they could try to improve [29]. The subjects were encouraged to self-monitor the actualization of the goals. The fulfillment of the goals was also discussed during the study visits. Weight was measured at every visit and the participants were encouraged to follow their weight regularly at home. The rate of weight loss not more than 0.5 to 1 kg per week was recommended. In addition, there were some voluntary group sessions, expert lectures and between-visit phone calls and letters. Three participants also received a very low-calorie diet (VLCD) for 2–5 weeks.

The participants were individually advised to increase their overall level of physical activity [29]. This was done and monitored by the nutritionist during the dietary counselling sessions and underlined by the study physicians at the annual visits. The participants were offered voluntary free of charge, supervised, individually tailored circuit-type moderate-intensity resistance training sessions in the gym to improve the functional capacity and strength of the large muscle groups of the body.

#### Control group

At baseline, the participants in the control group were given general verbal and written information about healthy lifestyle (diet and physical activity) and diabetes risk but no specific individually tailored advice was offered [29]. This was done either personally or in one group session (30 min to 1 h). The key messages were the same as for the intervention group participants: to reduce weight, increase physical activity and make qualitative changes in diet.

#### Post-intervention follow-up

During the post-intervention follow-up, all participants had a yearly visit with the study nurse [31]. These visits included the same measurement procedures as during the intervention period and were similar for all participants irrespective of their former group allocation. No detailed diet or exercise counselling was given.

### Study measures

Information about eating behavior dimensions was obtained by using the Three Factor Eating Questionnaire [10] translated to Finnish. The questionnaire was given as a paper version at every yearly visit during intervention and post-intervention follow-up phase to be filled out at home and returned via mail. The questionnaire contained 51 items, which were summed up into three scales: cognitive restraint of eating (21 items; measures conscious attempts to monitor and regulate food intake), disinhibition (16 items; measures uncontrolled eating in response to cognitive or emotional cues), and susceptibility to hunger (14 items; measures the extent to which respondents experience feelings of hunger in their daily living). Higher scores represented more cognitive restraint of eating, disinhibition, and perceived hunger. In addition to total score of cognitive restraint of eating, two additional scores, flexible (7 items) and rigid (7 items) restraint were calculated according to Westenhoefer [11] (Supplementary Table 2). In this study, term “dietary restraint” is used to describe all three: total cognitive, flexible and rigid restraint of eating. The Cronbach’s alpha’s were for total cognitive restraint *α* = 0.86, flexible restraint *α* = 0.74, rigid restraint *α* = 0.66, disinhibition *α* = 0.75 and susceptibility to hunger *α* = 0.76, indicating reasonable internal consistency.

Body weight was measured in light indoor clothing to the nearest 100 g at the baseline and at each annual visit.

### Statistical analysis

We present data for the measures of eating behavior dimensions and body weight changes up to nine years study period where data were available for 39 (76%) participants (for both eating behavior and body weight measures) in the intervention group, and 32 (70%) participants for eating behavior and 34 (72%) participants for body weight measures in the control group. Descriptive statistics, Pearson’s chi-squared and correlation tests, Mann–Whitney *U*-test and independent samples *t*-test were derived with IBM SPSS statistical software, version 27.0. Correlations were used to evaluate the associations between 1-year changes in eating behavior dimensions and 9-year changes in body weight (weight at year 9 – weight at baseline). All the tests were two-sided.

We analyzed Group × Time latent change scores using structural equation modeling (Mplus statistical software, version 8.4, Muthén and Muthén 1998–2020). We used full information maximum likelihood with robust standard errors and chi-square which accounts for missing values at random and includes all available data. The Group×Time latent change scores were tested to investigate the impact of the intervention on changes in the TFEQ scales and body weight (Wald test).

Effect sizes were investigated to analyze the possible difference between and within groups. Effect sizes were reported using Cohen’s *d* and were calculated as follows, both for the initial 1-year study period and the entire 9-year study period. The between-group effect sizes were calculated by subtracting the mean difference between the intervention and control groups at baseline from the mean difference between the intervention and control groups at 1-year or 9-years and the result was then divided by the pooled standard deviation of baseline measurements. The within-group effect sizes for both 1-year and 9-year study periods were calculated as follows: the mean change from the baseline to the 1-year measurement was divided by the combined (pooled) baseline and 1-year measurements’ standard deviation, and the mean change from the baseline to the 9-year measurement was divided by the combined baseline and 9-year measurements’ standard deviation. Effect size (between-group, within-group) *d* ≥ 0.2 was considered small, *d* ≥ 0.5 moderate and *d* ≥ 0.8 large [32].

**Fig. 1:**
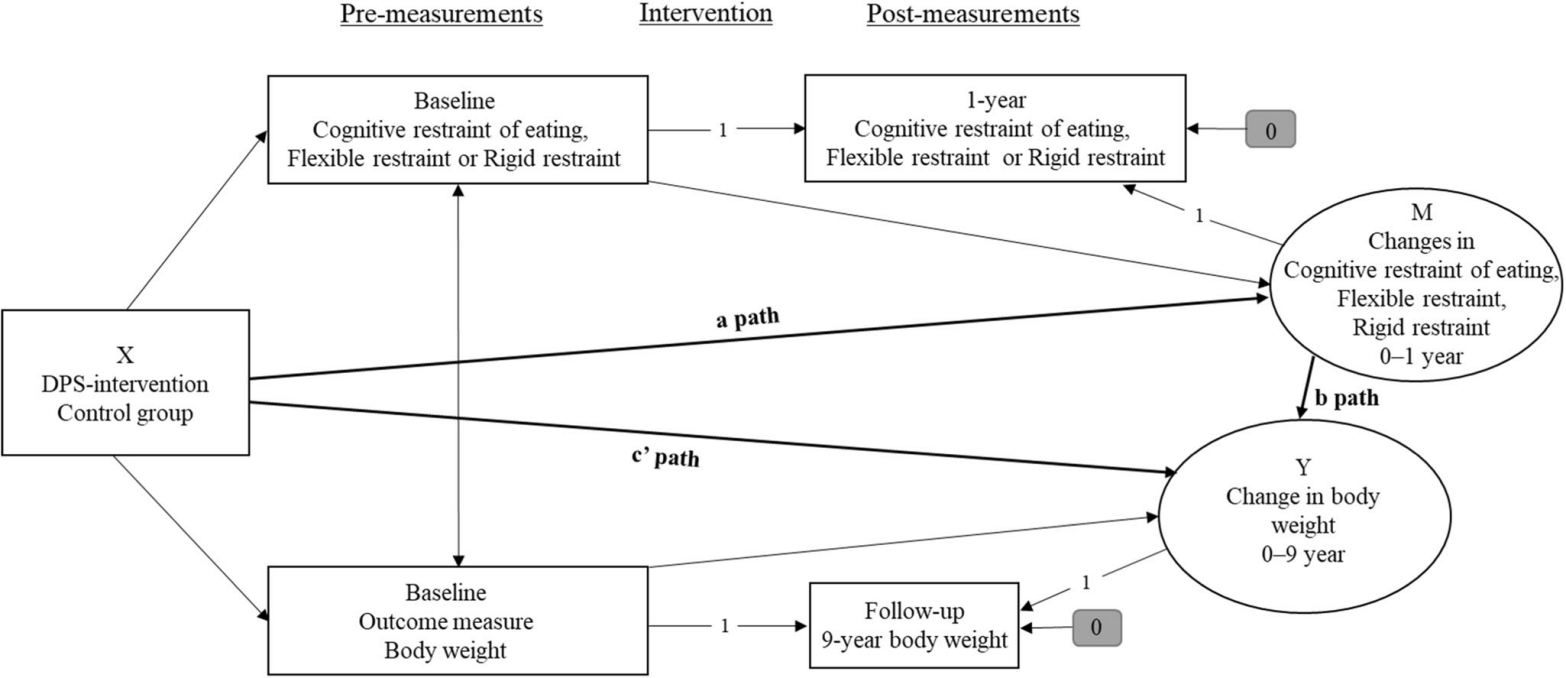
Mediation model. **a** The effect of the independent variable on the mediator. **b** The effect of the mediator on the dependent variable. **c**’ Direct effect of the independent variable on the dependent variable. The effects of sex and age were controlled for in the mediation model (not shown in the figure).

We analyzed mediation using structural equation modeling with full information maximum likelihood, which accounts for missing values at random and includes all available data. These simple mediation analyses (Fig. 1) were conducted to determine whether the effects of the intervention (independent variable) on 9-year change in body weight (dependent variable) were mediated by 1-year change in total cognitive restraint of eating or its rigid or flexible components (mediators). Disinhibition and hunger were not tested as mediators because their 1-year changes did not significantly differ between the intervention and control groups. Sex, age, baseline dietary restraint and baseline weight were controlled for in the analyses. The results were reported as the total, direct and indirect effects (i.e. regression coefficients and bias-corrected bootstrap 95% confidence intervals). The reported total effect (a × b + c’ in Fig. 1) illustrates the relationship between the intervention and weight change before adjustment for the change in dietary restraint. The indirect effect (a × b in Fig. 1) represents how much of the association between the intervention and weight change was explained by the dietary restraint change [33]. The indirect effect was considered statistically significant, if the 95% bias-corrected bootstrap confidence intervals (obtained using 5000 bootstrap resamples) did not include zero. The model fit was evaluated using the *χ*^2^ statistic, comparative fit index (CFI), and standard root mean square residual (SRMR). A non-significant *χ*^2^ (*p* > 0.05), CFI ≥ 0.95 and SRMR ≤ 0.08 were considered to indicate a good fit for the data [34].

## Results

The descriptive characteristics of the participants in the intervention and control groups did not differ at baseline (Table 1).

**Table 1:** Descriptive characteristics of the DPS intervention and control group participants in this sub-study at the baseline.

	Intervention group			Control group			*p*-value^a^
	*N*	Mean (SD)	Min-Max	*N*	Mean (SD)	Min-Max	
Age, year	51	53.9 (7.6)	39.8–64.6	47	53.2 (7.2)	40.9–64.8	0.631
Women, %		60.7	–		61.7	–	0.926^b^
Weight (kg)	51	86.2 (15.4)	60.5–120.2	47	87.7 (15.4)	61.4–131.5	0.641
BMI (kg/m^2^)	51	30.9 (4.1)	24.6–40.2	47	31.7 (5.1)	24.8–48.9	0.596
Cognitive restraint of eating, total^c^	51	9.0 (5.2)	0–20	46	9.6 (4.7)	1–20	0.491
Flexible restraint^c^	51	2.4 (2.1)	0–7	46	2.6 (1.9)	0–7	0.343
Rigid restraint^c^	51	2.7 (1.8)	0–7	46	3.0 (1.8)	0–7	0.558
Disinhibition^c^	51	5.6 (2.6)	1–15	46	6.2 (3.4)	1–15	0.543
Susceptibility to hunger^c^	51	4.2 (2.6)	1–11	46	4.6 (3.3)	1–14	0.815

*DPS* the Diabetes Prevention Study, *BMI* body mass index.

^a^Comparison between the intervention and control groups by using Mann–Whitney *U* Test or chi-square.

^b^Pearson chi-square.

^c^Scores of the Three Factor Eating Questionnaire subscale.

### Changes in body weight during the 9-year study period

At 9 years the intervention goal of weight reduction of 5% or more from the baseline was achieved by 43.6% of the participants in the intervention group as compared with 23.5% in the control group (*p* = 0.07). The mean percentage change in body weight at 9 years was −2.9% (SD 7.1) in the intervention and +0.8% (SD 5.8) in the control group (*p* = 0.05). Individual changes in body weight (in percentage) by group are shown in Supplementary Fig. 1.

**Fig. 2:**
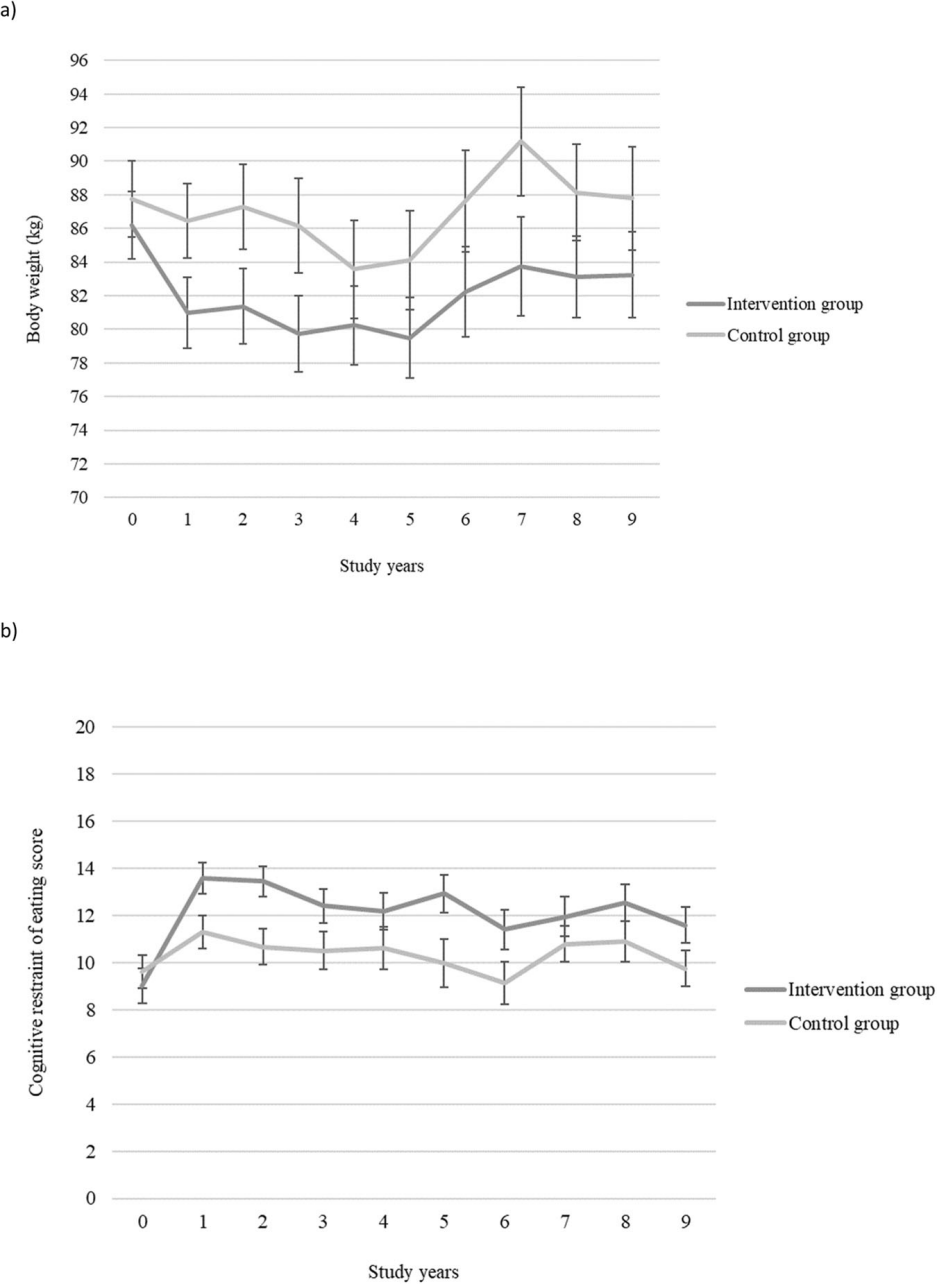
Changes in body weight and cognitive restraint of eating. Mean values and standard error bars of **a** body weight and **b** cognitive restraint of eating (the Three Factor Eating Questionnaire) during the 9-year study period in the Diabetes Prevention Study intervention and control groups.

There was a significant interaction effect (time x group) from baseline to year 9 in body weight (*p* = 0.046) indicating that the changes in weight differed between the groups (Table 2). Weight loss in the intervention group (−5.2 kg) was significantly larger than in the control group (−1.2 kg) during the first year of the study (*p* < 0.001) (Fig. 2). Furthermore, at 9 years weight loss was still significantly larger in the intervention group (−3.0 kg) compared with the control group (+0.1 kg) (*p* = 0.046) (Fig. 2). The between-group effect sizes for weight changes at year 1 and 9 were both in favor of the intervention group (Table 2).

**Table 2:** Means, standard deviations (SD) and the effects of the DPS intervention on eating behavior dimensions (scores of the Three Factor Eating Questionnaire subscales) and body weight (kg).

	Baseline mean (SD), Intervention *n* = 51, Control *n* = 47	1-year mean (SD), Intervention *n* = 47, Control *n* = 45	*p*-value^a^	Between group d^b^	9-year mean (SD), Intervention *n* = 39, Control *n* = 34	Wald’s test (df = 9) (*p*-value)^c^	Between group d^d^
Cognitive restraint of eating, total			<0.001	0.64		26.897 (0.002)	0.61
Intervention	9.00 (5.25)	13.58 (4.50)^e^			11.59 (4.78)		
Control	9.61 (4.67)^e^	11.31 (4.53)^e^			9.75 (4.30)^e^		
Flexible restraint			0.018	0.43		14.911 (0.093)	0.55
Intervention	2.37 (2.14)	4.09 (2.00)^e^			3.41 (2.07)		
Control	2.63 (1.85)^e^	3.57 (2.00)^e^			2.75 (2.11)^e^		
Rigid restraint			0.001	0.67		23.968 (0.004)	0.34
Intervention	2.73 (1.85)	4.36 (1.69)^de^			3.69 (1.79)		
Control	3.00 (1.76)^e^	3.50 (1.63)^de^			3.44 (1.56)^e^		
Disinhibition			0.949	0.04		8.533 (0.482)	0.06
Intervention	5.55 (2.60)	4.71 (2.87)^e^			3.64 (2.32)		
Control	6.17 (3.42)^e^	5.31 (3.27)^e^			4.47 (3.65)^e^		
Hunger			0.669	0.03		16.855 (0.051)	0.11
Intervention	4.24 (2.64)	3.36 (2.61)^e^			2.90 (2.54)		
Control	4.61 (3.26)^e^	3.79 (2.70)^e^			3.38 (2.67)^e^		
Weight			<0.001	0.22		17.164 (0.046)	0.16
Intervention	86.2 (14.32)	81.0 (14.45)			83.2 (15.88)		
Control	87.7 (15.44)	86.5 (14.87)			87.8 (17.96)		

*DPS* Diabetes Prevention Study.

^a^Investigates whether groups changed differently during the 1-year study period.

^b^1-year effect sizes between the intervention and control groups using corrected Cohen’s d.

^c^Investigates whether groups changed differently during the 9-year study period.

^d^9-year effect sizes between the intervention and control groups using corrected Cohen’s d.

^e^Eating behavior data missing: at baseline from one person in the control group, at 1 year from 2 persons in the intervention group, 3 persons in the control group; at 9 years from 2 persons in the control group.

### Changes in eating behavior dimensions during the 9-year study period

**Fig. 3:**
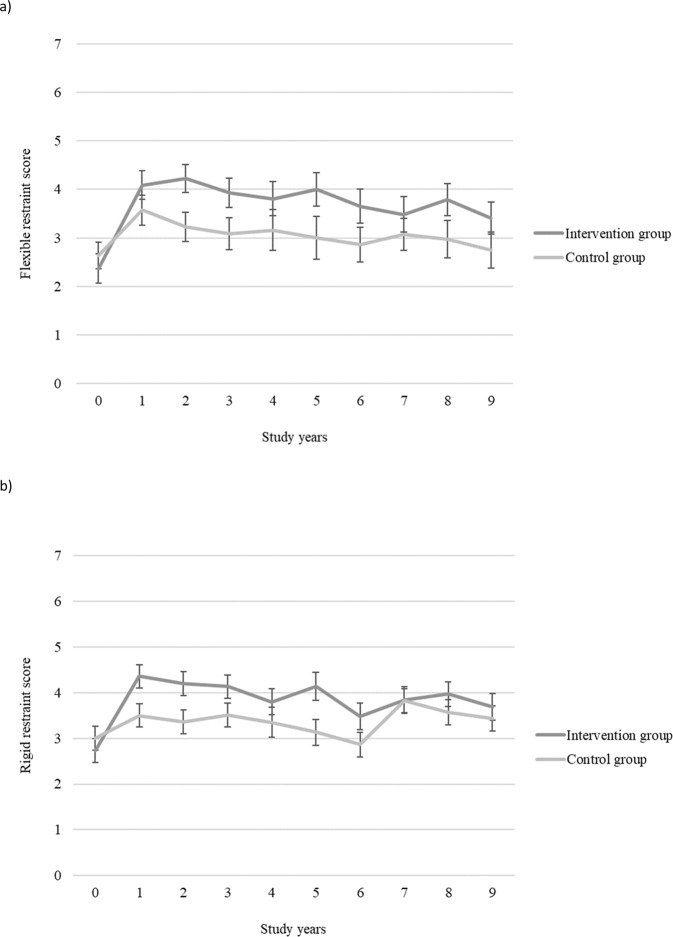
Changes in flexible and rigid restraint of eating. Mean values and standard error bars of **a** flexible restraint and **b** rigid restraint of eating (the Three Factor Eating Questionnaire) during the 9-year study period in the Diabetes Prevention Study intervention and control groups.

There was a significant interaction effect (time × group) from baseline to year 9 in total (*p* = 0.002) and in rigid (*p* = 0.004) restraint of eating indicating that the measures of these dimensions changed differently between the groups (Table 2). Total (*p* < 0.001) and rigid (*p* = 0.001) restraint increased significantly more in the intervention group compared with the control group during the first year of the study (Figs. 2, 3). The between-group effect sizes at year 1 and 9 were moderate (*d* > 0.5) for total and from moderate to small for rigid restraint in favor of the intervention group (Table 2). Within group effect sizes also showed larger changes in the intervention group compared with the control group from baseline to year 1 in total (intervention: *d* = 1.00, control: *d* = 0.38) and in rigid (intervention: *d* = 0.97, control: *d* = 0.30) restraint, where the change was large in the intervention group and small in the control group. Furthermore, from baseline to year 9 there was a moderate change in total cognitive restraint of eating in the intervention group but only negligible change in the control group (intervention: *d* = 0.58, control: *d* = 0.03) and a moderate change in rigid restraint in the intervention group but a small change in the control group (intervention: *d* = 0.56, control: *d* = 0.24).

Flexible restraint increased significantly more (*p* = 0.018) in the intervention group compared with the control group during the first year of the study (Fig. 3). Also, flexible restraint (*p* = 0.093) showed a trend in interaction effect from baseline to year 9 (Table 2). The between-group effect sizes at year 1 and 9 for flexible restraint were from small to moderate in favor of intervention group. Within group effect sizes also showed larger changes in the intervention group compared with the control group from baseline to year 1 in flexible restraint (intervention: *d* = 0.87, control: *d* = 0.51), where the change was large in the intervention group and moderate in the control group. From baseline to year 9 there was a moderate change in the intervention group but negligible change in the control group (intervention: *d* = 0.55, control: *d* = 0.03).

There was no significant interaction effect (time × group) from baseline to year 9 in disinhibition (*p* = 0.48) (Table 2). Susceptibility to hunger showed a trend in interaction effect from baseline to year 9 (*p* = 0.05). However, the change was not significantly different in the intervention group compared with the control group during the first year of the study. The between group effect sizes at year 1 and 9 for disinhibition and susceptibility to hunger were negligible.

Overall, in the intervention group the short-term changes in the measures of total cognitive, flexible and rigid restraint of eating were large and the long-term changes were moderate, whereas the changes were small or negligible in the control group, or in disinhibition or hunger in both groups.

### Associations between changes in eating behavior dimensions and body weight

The 1-year changes in total (*r* = −0.34, *p* = 0.004), rigid (*r* = −0.32, *p* = 0.007) and flexible (*r* = −0.23, *p* = 0.058) restraint of eating had moderate inverse correlations with the 9-year body weight change indicating that the more the measures of dietary restraint increased during the first year of the study, the more body weight decreased during the 9-year study period. The scatter plots of the correlations are shown in Supplementary Fig. 2.

### Dietary restraint scores mediating the effect of intervention on body weight change

Using three simple mediation models we investigated whether 1-year change in total cognitive restraint of eating or its rigid or flexible components mediated the effects of intervention on 9-year weight change (Table 3). The fit indices of all three mediation models indicated a good fit for the data (Table 3). The intervention was significantly related to a greater 1-year increase in total, flexible and rigid restraint (path a, Table 3), respectively. Increases in total and rigid restraint were related to a greater weight loss (path b, Table 3) and for flexible restraint this association showed a borderline trend for statistical significance (*p* = 0.057). Significant indirect effects (a × b, Table 3) on body weight change from baseline to year 9 were observed for 1-year change in total, flexible and rigid restraint, indicating that these changes statistically mediated the impact of the intervention on weight up to 9 years.

**Table 3:** Results from mediation models: total, direct and indirect (through 1-year change in dietary restraint) effects of the DPS intervention vs. control group on 9-year change in body weight.

Independent	Dependent	Mediator (baseline – 1 year)	a path (*p*-value)	b path (*p*-value)	Direct effect c′ (*p*-value)	Indirect effect a × b	Bootstrap results of indirect effect 95% CI	Total effect c (a × b + c′) (*p*-value)
Group (Intervention group, Control group)	Change in body weight (kg, baseline–9 y)^a^	Cognitive restraint of eating, total^b,e^	−2.874 (0.000)	−0.489 (0.008)	1.111 (0.455)	1.406	0.380, 3.194	2.517 (0.046)
		Flexible restraint^c,e^	−0.722 (0.033)	−0.817 (0.057)	2.015 (0.127)	0.590	0.002, 2.064	2.606 (0.041)
		Rigid restraint^d,e^	−0.988 (0.001)	−1.038 (0.008)	1.288 (0.354)	1.025	0.317, 2.298	2.313 (0.074)

*DPS* the Diabetes Prevention Study.

^a^Paths were controlled for age, sex and eating behavior and body weight baseline level (see Fig. 1 for paths a, b and c’).

^b^Chi-square = 3.890, df = 2, *p* = 0.143, CFI = 0.992, SRMR = 0.042.

^c^Chi-square = 1.334, df = 2, *p* = 0.513, CFI = 1.000, SRMR = 0.022.

^d^Chi-square = 2.893, df = 2, *p* = 0.235, CFI = 0.996, SRMR = 0.039.

^e^Score of the Three Factor Eating Questionnaire subscale.

## Discussion

This study offered a rare opportunity to study eating behavior dimensions and their associations with long-term weight changes in participants with IGT during the 9-year study period. We found that during the first year of the RCT, dietary restraint (i.e., total cognitive, flexible and rigid restraint of eating) increased and body weight decreased more in the intervention group receiving intensive and individually tailored lifestyle counselling compared with the control group receiving a standard health advice at baseline. These between-group differences remained up to nine years and the results from the mediation analyses suggested that early-phase increase in dietary restraint could have a role in long-term weight loss maintenance.

Our results on the effects of increase in dietary restraint on body weight are consistent with the few previous studies conducted in people with IGT [23, 25]. In the DPP, an increase in the restraint of eating during the initial six months of intervention as measured by DEBQ predicted the achievement of 7% weight loss target after 2.8-year intervention [25]. Also in the DELIGHT, a 1-year increase in flexible restraint as measured by extended version of TFEQ correlated with favorable changes in central obesity and fasting plasma glucose [23].

Furthermore, our study showed that the increase in total, rigid and flexible restraint during the first year statistically mediated the impact of DPS intervention on 9-year weight loss maintenance suggesting that all these restraint strategies could be associated with long-term weight loss maintenance when combined with intensive, individually tailored and professional lifestyle counselling. Also in the previous 3-year weight gain prevention study an increase in all three measures of dietary restraint was associated with decrease in weight at year three [18]. Instead, another intervention study investigating eating behavior dimensions as mediators of weight loss maintenance found that increase in flexible, but not rigid restraint, mediated a greater 24-month weight loss [27]. However, the participants of that study were all female and younger and study period substantially shorter than in the present study which might at least partly explain the different outcomes. Furthermore, Konttinen et al. found among participants with obesity who were treated at regular primary health care and were thus comparable to the control group of the DPS, that an increase in total cognitive restraint of eating measured with TFEQ predicted a greater 2-year weight loss, but not a 6- or 10-year weight loss [17].

Nevertheless, the concept of restrained eating is a controversial issue [19, 35]. This could be due to fact that different measures used to assess restraint of eating seem to capture different types of eaters [36, 37]. TFEQ has been shown to identify people who are motivated by health, successful to consistently control their food consumption, less likely to become disinhibited and more likely to lose or maintain weight over time as compared with restrained eaters identified by the Restraint Scale, another frequently used measure to assess dietary restraint [35, 38, 39]. Therefore, we emphasize that not any kind of dietary restraint would be beneficial. However, we suggest that in a professional lifestyle guidance it is possible to increase restraint of eating in a way that could be associated with successful long-term weight management.

Although we cannot define what were the exact intervention elements in the DPS that contributed to the current results, we can speculate they could have been related to self-regulation, a concept close to restraint of eating [19]. In the systematic review investigating psychological mediators of successful outcomes in obesity-related lifestyle change, self-regulation skills as well as autonomous motivation and self-efficacy emerged as the best predictors of beneficial weight and physical activity outcomes [21]. Emphasis should thus be put to interventions that combine lifestyle management with training of psychological skills, including self-regulation. Goal setting, self-monitoring and evaluation, problem solving, affect regulation and coping strategies in high-risk situations have been used as techniques to enhance self-regulation [19]. These techniques, especially goal setting, problem solving and self-monitoring and evaluation were integral part of the DPS intervention, too. These elements may have contributed to the strengthening of self-regulation skills, that was reflected as a better ability to cognitive restraint of eating which could have contributed to more successful weight loss maintenance. Consequently, training of self-regulation skills could be an important aspect to be emphasized in future lifestyle interventions. Although the data were collected 17−30 years ago the potentially effective intervention elements of the DPS are likely to be effective regardless of the time. Modern technology could offer means to conduct interventions utilizing these same elements even on a larger scale [40]. However, it should be ensured that such interventions are achievable and acceptable also in the vulnerable groups to not unintentionally widen health disparities. A recent review investigating explanations for the effectiveness of nutrition information interventions among adults with a low socioeconomic status (SES) emphasized tailored interventions including teaching of self-regulation skills as well as considering economic and social resources [41]. Furthermore, iterative design, use of visual and multimedia elements and social support have been identified as facilitators of eHealth interventions in groups with low SES [40].

The nine years study duration along with the initial RCT setting are the greatest strengths of the present study. The relatively low attrition, in turn, is a marker of high commitment to the intervention. Furthermore, the use of mediation analyses allowed us to gain more detailed knowledge on the potential role of eating behavior dimensions in the long-lasting body weight decrease among the DPS participants. Moreover, the difference in the long-term weight change between the groups was present after nine years and was comparable or even somewhat greater than the long-term weight reduction reported earlier in the entire DPS study population [42]. This earlier long-term report on the DPS showed that the risk of developing T2DM was still 37% smaller in the intervention group than in the control group even after 13 years. Besides better glucose values the intervention group had also healthier diet and other health benefits, e.g., lower blood pressure and triglycerides compared with the control group [42].

The limitations of the study were a relatively small number of participants as well as varied duration of the intervention period. The DPS study participants were volunteers willing to take part in a long-term trial, which can indicate that they were more health-conscious and motivated compared with the general population. The participants were mostly middle-aged, and the results may be different among younger or older population. In the mediator model, it was assumed that the change in dietary restraint affects the change in body weight. Because of the temporal process of weight loss maintenance (first weight loss, then weight maintenance), the changes in eating behavior dimensions and body weight partially overlapped in the model. However, the overlapping period was only one year as compared to nine years in body weight change. Nevertheless, we cannot rule out that part of the association between the change in dietary restraint and the change in body weight might have happened also the other way round, i.e., the change in body weight might have affected the change in dietary restraint.

To conclude, lifestyle intervention with intensive and individually tailored, professional counselling had long-lasting effects on cognitive restraint of eating and body weight in middle-aged participants with overweight and IGT. The results of the mediation models suggest that early phase increase in cognitive restraint of eating could have a role in long-term weight loss maintenance which may confer various health benefits, including reduced risk of T2DM.

## Supplementary information

Supplementary Table 1.

Supplementary Table 2.

Supplementary Figure 1.

Supplementary Figure 2.

## Data Availability

The data are subject to national data protection laws. Therefore, data cannot be made freely available in a public repository. However, data can be requested through an individual project agreement. To obtain permission reasonable requests should be addressed to the corresponding author.
