# Supplementary material for: Eating behavior dimensions and 9-year weight loss maintenance: a sub-study of the Finnish Diabetes prevention study
Source: Int J Obes (Lond). 2023 May 6;47(7):564–73. doi: 10.1038/s41366-023-01300-w (PMC10299913; doi:10.1038/s41366-023-01300-w)
Supplement: Supplementary file 1 — Supplementary Table 1. [file 41366_2023_1300_MOESM1_ESM.docx]

**Supplementary Information**

**Supplementary Table 1.** Descriptive baseline characteristics of the study completers and drop-outs in the substudy of DPS

|  | Completers | | | Drop-outs | | |  |
| --- | --- | --- | --- | --- | --- | --- | --- |
|  | N | Mean (SD) | Min-Max | N | Mean (SD) | Min-Max | p-value ^a^ |
| Age, year | 74 | 54.2 (6.6) | 41.2–64.8 | 24 | 51.5 (9.2) | 39.8–64.6 | 0.183 |
| Women, % |  | 58.1 | - |  | 70.8 | - | 0.266^b^ |
| Weight (kg) | 74 | 86.4 (15.6) | 60.5–131.5 | 24 | 88.6 (12.2) | 70.4–120.2 | 0.306 |
| BMI (kg/m^2^) | 74 | 30.9 (4.7) | 24.6–48.9 | 24 | 32.4 (3.9) | 26.4–40.4 | 0.061 |
| Cognitive restraint of eating, total ^c^ | 74 | 9.6 (5.1) | 0–20 | 23 | 8.4 (4.4) | 2–18 | 0.264 |
| Flexible restraint ^c^ | 74 | 2.5 (2.0) | 0–7 | 23 | 2.4 (1.9) | 0–7 | 0.757 |
| Rigid restraint ^c^ | 74 | 2.9 (1.9) | 0–7 | 23 | 2.7 (1.6) | 0–6 | 0.639 |
| Disinhibition ^c^ | 74 | 5.9 (3.1) | 1–15 | 23 | 5.7 (2.9) | 2–12 | 0.851 |
| Susceptibility to hunger ^c^ | 74 | 4.5 (3.0) | 1–14 | 23 | 4.0 (2.7) | 1–10 | 0.462 |

^a^ Comparison between completers and drop-outs by using Mann-Whitney U Test or chi-square, ^b^Pearson chi-square, ^c^ scores of the Three Factor Eating Questionnaire subscale, DPS = the Diabetes Prevention Study, BMI = Body Mass Index.
