# Supplementary material for: Eating behavior dimensions and 9-year weight loss maintenance: a sub-study of the Finnish Diabetes prevention study
Source: Int J Obes (Lond). 2023 May 6;47(7):564–73. doi: 10.1038/s41366-023-01300-w (PMC10299913; doi:10.1038/s41366-023-01300-w)
Supplement: Supplementary file 2 — Supplementary Table 2. [file 41366_2023_1300_MOESM2_ESM.docx]

**Supplementary Information**

**Supplementary Table 2.** The items for flexible and rigid restraint of eating subscales in the Three Factor Eating Questionnaire (Westenhoefer, 1991) where scored options are underlined.

| **Flexible restraint** |
| --- |
| 4. When I have eaten my quota of calories, I am usually good about not eating any more (true–false) |
| 6. I deliberately take small helpings as a means of weight control (true–false) |
| 18. While on a diet, if I eat food that is not allowed, I consciously eat less for a period of time to make up for it (true–false) |
| 28. I consciously hold back at meals in order not to gain weight (true–false) |
| 35. I pay a great deal of attention to changes in my figure (true–false) |
| 42. How conscious are you of what you are eating? (not at all–slightly–moderately–extremely) |
| 48. How likely are you to consciously eat less than you want? (unlikely–slightly unlikely–moderately likely–very likely) |
| **Rigid restraint** |
| 14. I have a pretty good idea of the number of calories in common food (true–false) |
| 32. I count calories as a conscious means of controlling my weight (true–false) |
| 37. How often are you dieting in a conscious effort to control your weight? (rarely–sometimes–usually–always) |
| 38. Would a weight fluctuation of 5 lb affect the way you live your life? (not at all–slightly–moderately–very much) |
| 40. Do feelings of guilt about overeating help you to control your food intake? (never–rarely–often–always) |
| 43. How frequently do you avoid “stocking up” on tempting foods? (almost never–seldom–usually–almost always) |
| 44. How likely are you to shop for low calorie foods? (unlikely–slightly unlikely–moderately likely–very likely) |
