# Supplementary material for: Eating behavior dimensions and 9-year weight loss maintenance: a sub-study of the Finnish Diabetes prevention study
Source: Int J Obes (Lond). 2023 May 6;47(7):564–73. doi: 10.1038/s41366-023-01300-w (PMC10299913; doi:10.1038/s41366-023-01300-w)
Supplement: Supplementary file 3 — Supplementary Figure 1. [file 41366_2023_1300_MOESM3_ESM.pdf]

Supplementary Figure 1. Individual changes in body weight (in percentage) in the intervention and control groups during the 9 years study period.

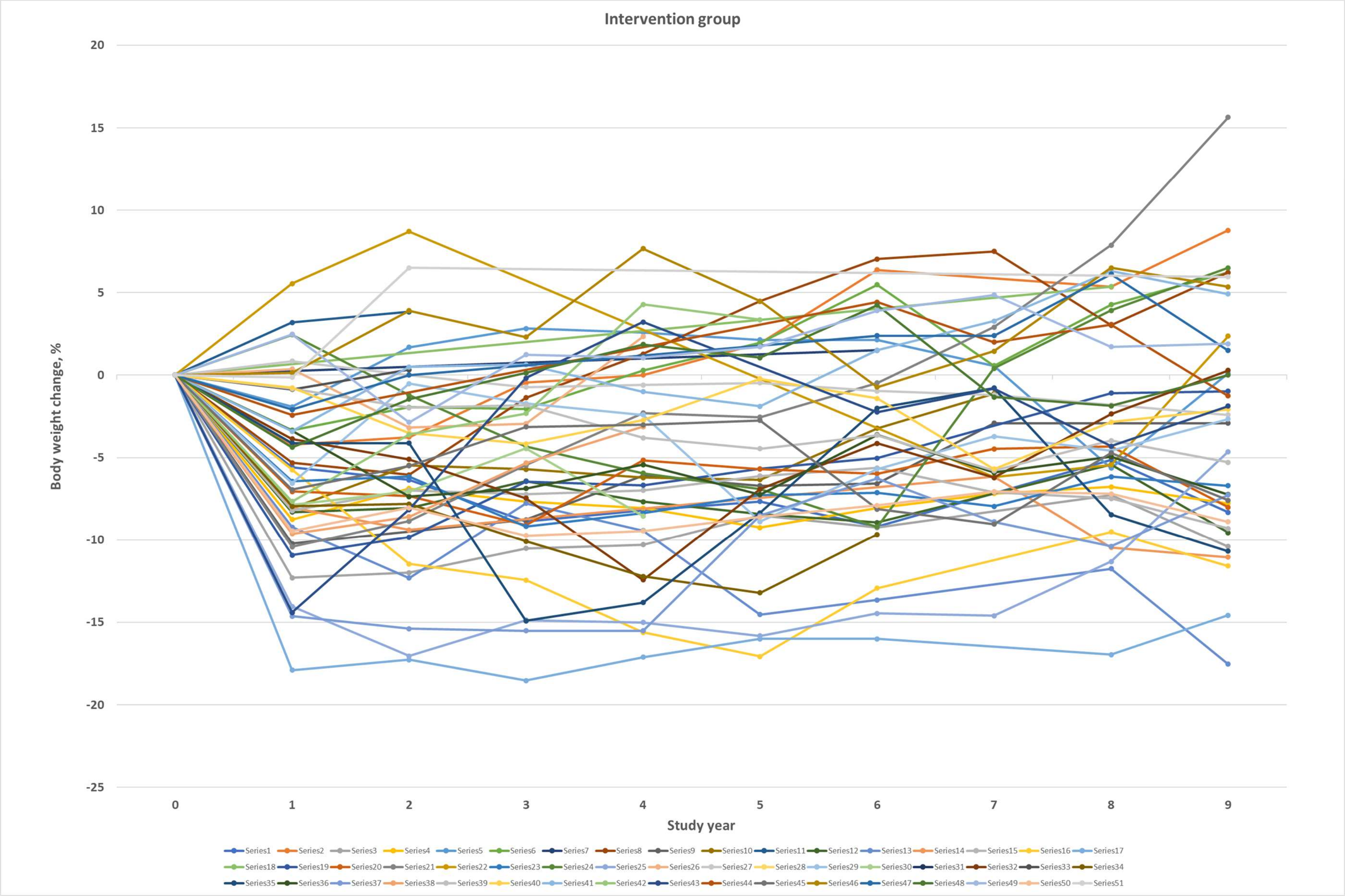

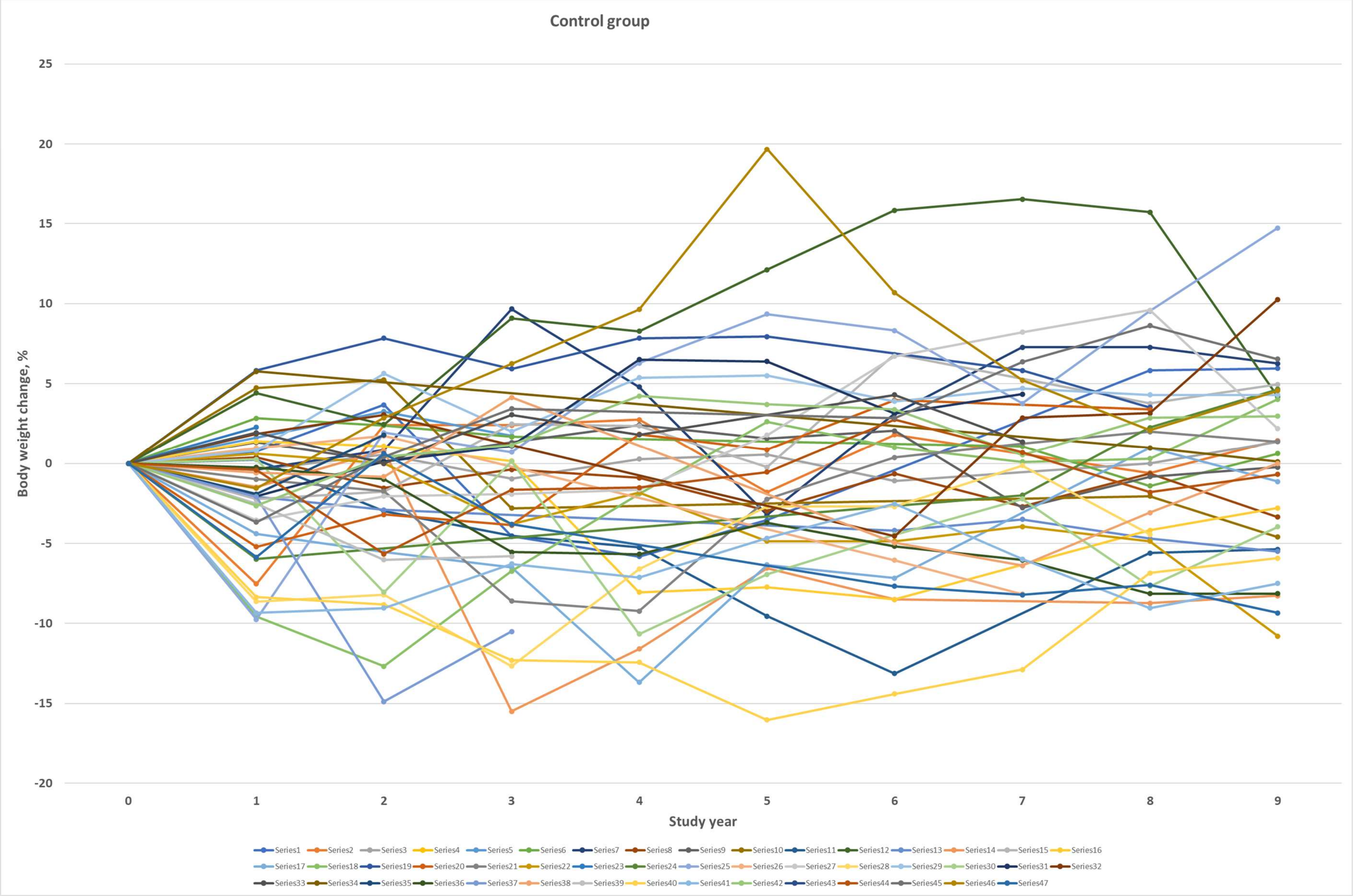
Control group
Body weight change, %
25
20
15
10
5
0
-5
-10
-15
-20
0
1
2
3
4
5
6
7
8
9
Study year
Series1 Series2 Series3 Series4 Series5 Series6 Series7 Series8 Series9 Series10 Series11 Series12 Series13 Series14 Series15 Series16
Series17 Series18 Series19 Series20 Series21 Series22 Series23 Series24 Series25 Series26 Series27 Series28 Series29 Series30 Series31 Series32
Series33 Series34 Series35 Series36 Series37 Series38 Series39 Series40 Series41 Series42 Series43 Series44 Series45 Series46 Series47
