# Supplementary material for: Eating behavior dimensions and 9-year weight loss maintenance: a sub-study of the Finnish Diabetes prevention study
Source: Int J Obes (Lond). 2023 May 6;47(7):564–73. doi: 10.1038/s41366-023-01300-w (PMC10299913; doi:10.1038/s41366-023-01300-w)

**Supplementary Information**

**Supplementary Figure 2.** The scatter plots of correlations between (a) the 1-year change in total cognitive restraint of eating and the 9-year change in body weight (r= -0.34, p=0.004), (b) the 1-year change in rigid restraint and the 9-year change in body weight (r= -0.32, p=0.007), and (c) the 1-year change in flexible restraint and the 9-year change in body weight (r= -0.23, p=0.058).

**a)**


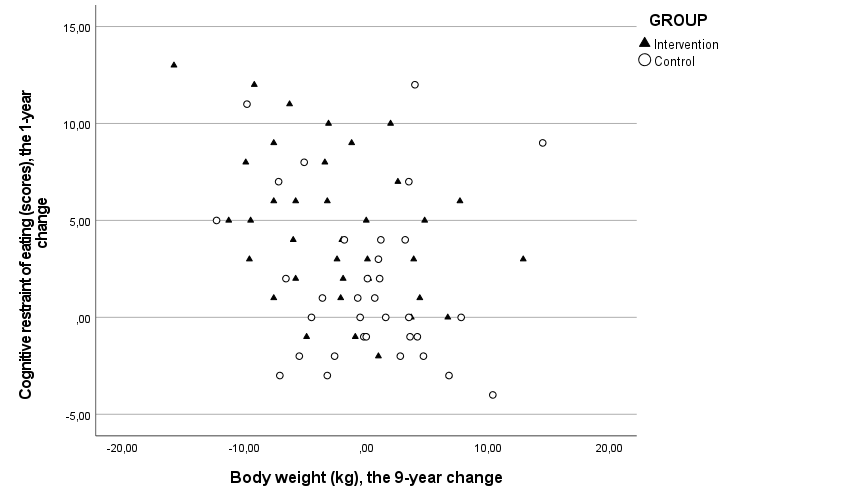


**b)**


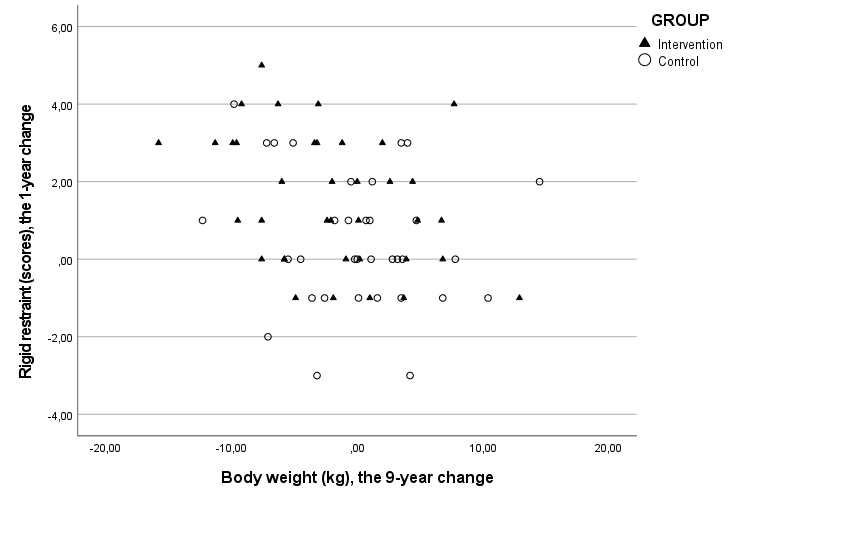


**c)**


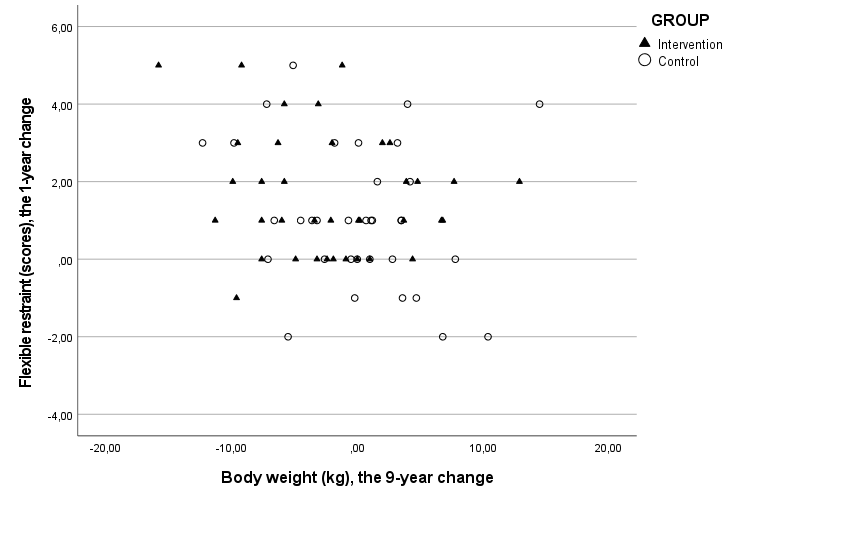

Supplement: Supplementary file 4 — Supplementary Figure 2. [file 41366_2023_1300_MOESM4_ESM.docx]
